# Information-Theoretical Analysis of Team Dynamics in Football Matches

**DOI:** 10.3390/e27030224

**Published:** 2025-02-21

**Authors:** Yi-Shan Cheng, Acer Yu-Chan Chang, Kenji Doya

**Affiliations:** 1 Okinawa Institute of Science and Technology, Okinawa 9040412, Japan; doya@oist.jp; 2 Department of Psychology, College of Contemporary Psychology, Rikkyo University, Tokyo 1718501, Japan; acercyc@rikkyo.ac.jp

**Keywords:** team dynamics, causal emergence, information theory, graph theory, football analytics, collective behavior

## Abstract

Team dynamics significantly influence the outcomes of modern football matches. This study employs an information-theoretical approach, specifically causal emergence, combined with graph theory to explore how team-level dynamics arise from complex interactions among players, utilizing tracking data from 34 J-League matches. We focused on how collective behaviors arise from the interdependence of individual actions, examining team coordination and dynamics through player positions and movements to identify emergent properties. Specifically, we selected relative distance to the field’s center, center of mass (CoM) and clustering coefficients based on velocity similarity and inverse distance as macroscopic features to capture the key aspects of team structure, coordination, and spatial relationships. Relative distance and CoM represent the collective positioning of the team, while clustering coefficients provide insights into localized cooperation and movement similarity among the players. The results indicate that average causal emergence with relative distance and CoM as a macroscopic feature across entire games shows a strong correlation with differences in ball possession rate between home and away teams. In contrast, clustering coefficients based on inverse distance and velocity similarity showed moderate to weak correlations with ball possession rate, indicating that these metrics may capture localized interactions that are less directly tied to team-level emergent behavior compared to CoM. Additionally, relative distance and CoM as macroscopic features yield higher causal emergence in attacking phases than in defending phases before shooting, suggesting that the collective positioning of players may play a more significant role in facilitating successful attacks than in defensive stability. This study offers a novel perspective on team coordination in football, suggesting that effective team coordination may be characterized by emergent patterns arising from collective positioning. These findings have practical implications for understanding coordinated team behaviors and inform coaching and performance analysis focused on enhancing team dynamics.

## 1. Introduction

In modern football, team dynamics play a crucial role in determining the outcome of matches [1,2]. Understanding how players interact on the field, cooperate, and execute tactical plans is key to optimizing team performance. Recent advances in data analytics and tracking technologies have enabled detailed analysis of player movements and interactions [3], yet quantifying teamwork remains a complex challenge.

Despite the availability of substantial amounts of player tracking data, current methods often fall short in capturing the complexity of team cooperation. Traditional metrics focused on individual players’ physical ability [4], performance [5], and the training [6] or coaching [7], which may overlook the emergent properties that arise from the complex interactions within a team. These methods fail to adequately reflect the dynamic, collective behaviors that contribute to team success, which are often greater than the sum of the individual players’ actions. Therefore, there is a need for new approaches that can accurately represent the collective coordination and emergent dynamics that underpin successful teamwork.

Emergence, a key concept in complex systems, describes how macroscopic behaviors or properties arise from the interactions of individual components, often resulting in patterns that cannot be fully understood by analyzing each component in isolation. For example, Seth demonstrated that migratory bird flocks do not act as mere aggregates of individual birds but form cohesive, coordinated groups where shared directionality and collective decision making emerge from interactions between individuals [8]. Collective behaviors are observed in the swarming of soldier crabs [9], fish schools [10] and other natural group phenomena. These examples illustrate emergence as a property of systems in which behaviors at the macroscopic level arise from the interactions of individual microscopic elements, resulting in patterns that cannot be fully understood by examining each component in isolation [11,12].

Causal emergence builds upon traditional emergence, which describes how macroscopic properties arise from the interactions of individual components in a system. Emergence, while providing descriptive insights, lacks a quantitative framework to evaluate how macroscopic properties influence system behavior. Causal emergence addresses this limitation by providing a method to compare macro-level and micro-level models in terms of their ability to explain or predict system dynamics. Hoel et al. [13] introduced causal emergence as a framework for analyzing when macro-level models capture causal patterns that are not evident at the micro-level. Their work has been applied in various domains to identify conditions under which macro-level properties provide additional explanatory value. Yuan et al. [14] reviewed recent advancements in causal emergence theory, summarizing its theoretical and applied developments in analyzing complex systems. In football, macro-level properties such as formations, passing networks, and coordinated movements are critical to understanding match outcomes. These properties are difficult to fully capture through micro-level analysis of individual players’ actions. This study applies causal emergence theory to investigate how macro-level structures contribute to team coordination and performance, offering insights that cannot be obtained from micro-level data alone.

To analyze causal emergence within team dynamics, we adopt the framework developed by Rosas et al. [15], which uses information-theoretic methods to quantify emergent behaviors. This framework applies Partial Information Decomposition (PID) to decompose shared information between variables into unique, redundant, and synergistic components [16]. Synergy represents information that arises from the joint state of multiple variables and cannot be assigned to any single variable, while redundancy reflects information that is shared across variables and can be attributed to multiple sources. By examining both synergy and redundancy, this framework provides a detailed view of how team-level coordination emerges from micro-level interactions among football players.

In this study, we also consider network interaction generated from graph theory to analyze team dynamics. These metrics emphasize relational dynamics and quantify structural and functional properties within the team’s network, providing insights into coordination patterns. Network science has been applied in various studies to analyze the structure and performance of football teams. For example, graph theory has been used to evaluate the passing networks of teams during the FIFA World Cup [17], demonstrating that high clustering coefficients were associated with successful teams, particularly in possession-dominant styles. Similarly, Buldú et al. applied network metrics to analyze team coordination, showing how the clustering coefficient can highlight the ability of players to maintain ball circulation and control [18]. These studies underscore the importance of network-based metrics in capturing the collective properties of teams.

By incorporating weighted clustering coefficients into football analysis [19], this study aims to highlight these network metrics not merely as descriptive indicators of team performance but as potential emergent properties arising from the interactions of individual players. Specifically, clustering coefficients, derived from micro-level features of velocity similarity and physical distance between players, are treated as collective features that reflect the coordinated behaviors of the team as a whole. This approach shifts the focus from evaluating how these metrics correlate with team success to investigating whether they capture emergent dynamics that cannot be fully explained by individual-level attributes alone. By doing so, we aim to explore the extent to which these metrics represent higher-order properties of team coordination and their role in the causal emergence of team performance.

The primary objective of this study is to investigate how causal emergence reveals macro-level team coordination arising from micro-level player interactions in football. Specifically, we explore how variations in causal emergence may be influenced by both synergy and redundancy, acknowledging their potential combined contributions to emergent team behaviors. This approach provides a framework for analyzing how team dynamics emerge, offering a deeper understanding of the mechanisms driving effective coordination.

This research merges causal emergence theory with network-based metrics, aiming to identify the role of emergent properties in team performance. By integrating information-theoretic tools with clustering coefficients, we analyze the interplay between micro-level interactions and macro-level patterns, contributing to both sports analytics and the broader field of complex systems research.

This study addresses the following key research questions:

1. How does causal emergence reflect emergent team coordination in football?
2. Can weighted clustering coefficients, derived from velocity similarity and physical distance, be interpreted as emergent properties of collective team behavior?
3. What role do emergent properties play in shaping successful team performance in terms of coordinated movements and match outcomes?

The structure of this paper is organized as follows: Section 2 introduces the theoretical framework and methodology. Section 3 presents the results, focusing on the relationship between causal emergence and possession rate, including different choices of macro-level features Section 4 discusses the implications of these findings for understanding team dynamics and their broader applications. Finally, Section 5 concludes with a summary with key insights and suggestions for future research directions.

## 2. Materials and Methods

### 2.1. Data Preparation

The dataset used in this study was provided by DataStadium Inc., Tokyo, Japan, under an agreement with the J-League. These proprietary tracking data include 34 J-League matches from the 2022 season, with usage approved by DataStadium Inc. and in compliance with all legal and ethical standards.

#### Preprocessing

The original player tracking data include positional information recorded at 25 Hz. The data were first down-sampled to 1 Hz. Since velocity information was not provided directly, the x and y velocity components for each player were calculated as the difference in position coordinates between consecutive seconds.

To determine the start and end frames of each half, event data were utilized. Event data included timestamps for match-specific actions such as passes, shots, fouls, and substitutions, providing a temporal reference to segment the tracking data. This ensured that only the frames corresponding to each half were extracted, allowing for precise alignment of positional data with gameplay phases.

During player substitutions, event data were also used to ensure that positional data for ten outfield players from each team remained available, excluding goalkeepers due to their unique role and restricted movement range. Furthermore, data from match periods involving red card incidents were excluded to maintain consistency with ten players per team for subsequent analyses.

### 2.2. Causal Emergence

To measure causal emergence in this study, we adopted the framework introduced by Rosas et al. [15]. This framework considers causal emergence as grounded in the unique causal power exhibited by macroscopic features $V_{t}$ at time t in predicting the system’s future state $X_{t^{\prime }}$ at time t′. To capture the concept of unique causal power, this framework utilizes Partial Information Decomposition (PID). PID, introduced by Williams and Beer, decomposes the total information that a set of source variables provides about a target variable into the following distinct components: unique, redundant, and synergistic information [16]. For example, consider two variables $X_{1}$ and $X_{2}$ as sources of information about a target variable Y. PID decomposes the total mutual information between $\left(X_{1},X_{2}\right)$ and Y into the following: unique information, which is the part of Y that is predicted only by $X_{1}$ or only by $X_{2}$; redundant information, which represents the information about Y that is shared by both $X_{1}$ and $X_{2}$; and synergistic information, which captures the information about Y that is not present in either $X_{1}$ or $X_{2}$ individually but emerges from their combined state.

In the context of causal emergence, unique information captures the part of the macroscopic feature $V_{t}$ that provides predictive power beyond what is available at the next future microscopic state $X_{t^{\prime }}$. Therefore, the formal definition of causal emergence, as proposed by Rosas et al. [15], is as follows: In a system described by $X_{t}$, a supervenient feature $V_{t}$ exhibits causal emergence of order l if:

(1) $${\mathrm{Un}}^{\left(l\right)}\left(V_{t};X_{t^{\prime }}|X_{t}\right)>0$$

where ${\mathrm{Un}}^{\left(l\right)}\left(V_{t};X_{t^{\prime }}|X_{t}\right)$ represents the unique information of $V_{t}$ about $X_{t^{\prime }}$ given $X_{t}$. at order l. Order l specifies the level of decomposition used in the analysis and determines the maximum subset size of microscopic variables considered when evaluating causal emergence. This definition captures the idea that a macroscopic feature $V_{t}$ exhibits causal emergence if it contains predictive information about the system’s future that is not available in any subset of its microscopic components $X_{t}$.

In practical application, to address computational limitations, a practical criterion of causal emergence was used, comparing the mutual information $I\left(V_{t};V_{t^{\prime }}\right)$ at the macroscopic level with the aggregate $\Sigma_{i\in s}I\left(X_{t}^{i};V_{t^{\prime }}\right)$ across microscopic features.

Therefore, the practical criteria used to measure causal emergence Ψ as proposed by Rosas et al. [15], is defined as follows:

(2) $$\Psi_{t,t^{\prime }}^{l}\left(V\right)≔I\left(V_{t};V_{t^{\prime }}\right)-\Sigma_{i\in s}{I(X}_{t}^{i};V_{t^{\prime }}),$$

where $I\left(V_{t};V_{t^{\prime }}\right)$ represents the mutual information between the macroscopic feature V at two time steps, t and a step after $t^{\prime }$, but not all of the time steps before $t^{\prime }$. The second term, $\Sigma_{i\in s}I(X_{t}^{i};V_{t^{\prime }})$, is the sum of the mutual information between each microscopic feature $X^{i}$ at time t and the macroscopic feature V at the subsequent time t′. Here, i indexes each microscopic feature within the set s of all microscopic feature s, allowing us to assess the combined contribution of all microscopic components relative to the macroscopic state. The practical criterion is particularly useful for empirical studies because it provides a computable metric that preserves the core concept of causal emergence. To estimate the mutual information terms here, we used the Gaussian mutual information estimator implemented in the publicly available toolbox provided by Rosas et al. [15]. This estimator assumes that variables are jointly Gaussian-distributed and provides an efficient closed-form computation of mutual information [20].

Note that we restricted our analysis to order l=1, meaning we only considered the contributions of individual microscopic features in explaining the predictive power of the macroscopic feature. Higher orders of l (e.g., l=2 or greater) that consider combinations of two or more microscopic features were not included in this study.

In the original proposal by Rosas et al. [15], causal emergence is identified when Ψ is positive, indicating that the macroscopic level provides sufficiently greater predictive power regarding the future state than the sum of the contributions from the microscopic components. Positive values of Ψ suggest that the macroscopic state captures emergent patterns and interactions that cannot be fully explained by the collective contributions of individual microscopic features. However, in our study, we also considered cases where Ψ is negative, focusing on the dynamics of causal emergence rather than only its positive manifestations.

Real-world systems, including team dynamics, often exhibit substantial redundancy, where overlapping information between system components contributes to the robustness and coordination of the system. Redundancy can lead to negative values of Ψ, reflecting scenarios where the macroscopic state retains less predictive power to the future macro-state than the total contributions of the predictive power of individual components to the future macro-state. Importantly, in the context of the current study, redundancy does not imply a lack of collective behavior but instead highlights the shared information among players that supports the overall system’s functionality. This shared information can enhance team coordination and resilience, even when the predictive power of individual components to the future macro-state exceeds that of the macroscopic feature.

Understanding redundancy is critical for interpreting collective behaviors in team sports. It provides insight into how shared information among players contributes to robust coordination and adaptability under various game conditions. By examining both positive and negative values of Ψ, this study aims to capture the full spectrum of causal emergence dynamics, providing a more comprehensive understanding of how team-level behaviors arise from the interplay of individual interactions.

#### Microscopic and Macroscopic Feature Selection

**Microscopic Feature Selection**

**Figure 1:**
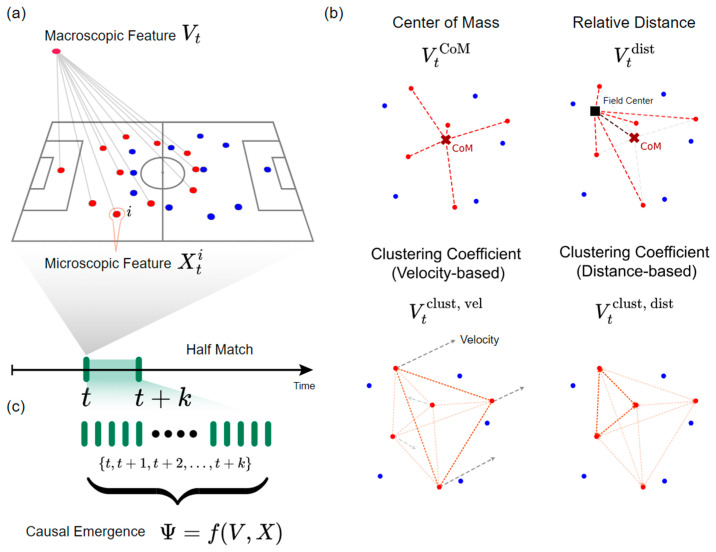
Framework for macroscopic feature selection and causal emergence analysis in football team dynamics. (**a**) Generation macroscopic feature $V_{t}$ from microscopic features $X_{t}^{i}$. (**b**) Schematic of macroscopic features based on CoM ($V_{t}^{\mathrm{CoM}}$), which was generated from players’ positions as microscopic features; distance from CoM to the field’s center ($V_{t}^{\mathrm{dist}}$), which was generated from players’ distances to the field’s center as microscopic features; clustering coefficient of velocity similarity ($V^{\mathrm{clust},\;\mathrm{vel}}$), which was generated from players’ velocities as microscopic features; and inverse distance ($V^{\mathrm{clust},\;\mathrm{dist}}$), which was generated from players’ positions as microscopic features. The red and blue dots presented the players’ positions in different teams and the lines are the distances between two objects. (**c**) Causal emergence Ψ analysis with interval {t,t+k}.

In the context of our causal emergence analysis, the selection of appropriate microscopic variables is essential as they form the foundation for deriving macroscopic features and identifying emergent team behaviors. For this study, we chose player positions, velocities, and distance to the field center as our primary microscopic features (illustrated in Figure 1). Player positions, represented by their x and y coordinates on the field, capture the spatial configuration of the team and are used to derive the macroscopic feature center of mass and the clustering coefficient of inverse distance from network analysis (see below). Player velocities, derived from the change in position over time, reflect the dynamic aspects of individual player movements and are used to compute the macro feature, the clustering coefficient of velocity similarity. Additionally, we included each player’s distance to the field’s center to account for their spatial relationship to a key reference point on the field; this is aggregated to form the macroscopic feature, relative distance. These microscopic variables were chosen because they directly represent the actions and spatial context of individual players. They provide a detailed, granular perspective on individual contributions before we aggregate them into team-level macroscopic features to explore emergent properties.

**Macroscopic Feature Selection**

In this study, the selection of macroscopic features and their corresponding microscopic variables, as presented in Figure 1, is pivotal for capturing the emergent team behaviors that arise from the complex interactions between individual players. These features serve as aggregated representations of team-level dynamics, providing insights that are not evident from analyzing individual components alone. We focus on two main categories of macroscopic features: aggregate positioning metrics and network interaction metrics.

**Aggregate Positioning Metrics**

Aggregate positioning metrics emphasize the collective positioning and spatial organization of the team. These features are derived from geometric configurations of player positions and capture the overall team structure. The selected metrics in this category include the following:

**Center of Mass (**$V^{\mathrm{CoM}}$**)**:

The microscopic features used to compute the center of mass are the x and y coordinates of all players on the field. The macroscopic feature is obtained by averaging these individual player positions, resulting in a two-dimensional representation of the team’s spatial structure. This approach captures overall team positioning, offering a direct measure of collective spatial dynamics.

**Relative Distance (**$V^{\mathrm{dist}}$**)**:

For each player, the distance to the field’s center was used as a microscopic feature, while the distance from the CoM to the field center served as a one-dimensional macroscopic feature. This representation highlights the team’s spatial orientation relative to a fixed reference point.

**Network Interaction Metrics**

Network interaction metrics are derived from graph theory and emphasize the relational dynamics and interactions among players. Specifically, we focus on the clustering coefficient, a metric that measures the degree of local clustering or interconnectedness within a network [21]. To construct graphs, we consider each player as a node and take two approaches to defining the edges of the graph: velocity-based similarity and distance-based proximity.

**Velocity-Based Edge Construction** **:**

To capture the degree of local coordination based on the players’ velocity, we calculated the clustering coefficient for each second from player’s velocity components in the x and y directions as the microscopic features. Each player’s velocity and movement direction were derived by calculating the distance and angle between consecutive positions. For each second, a weighted, undirected graph was constructed with nodes representing individual players and edges representing the relationships between the players. These relationships were weighted based on relative velocity and angular differences between the players, allowing us to reflect synchronization in player movements.

The weight of an edge between two players u and v was calculated as follows:

(3) $$Weight\left(u,v\right)=S_{u}\times S_{v}\times \left(cos\left(\Delta \theta \right)+1\right),$$

where $S_{u}$ and $S_{v}$ represent the players’ velocity, and Δθ is the difference in their movement angles. This approach ensures that two players with higher movement velocities and similar moving directions are assigned higher weights to their edges, indicating greater synchronization.

**Distance-Based Edge Construction** **:**

To capture spatial cooperation among players, we used each player’s Euclidean coordinates as the microscopic features and computed the clustering coefficient based on inverse distance metrics as the macroscopic feature. This approach assigns stronger edges to players who are physically closer, reflecting a higher likelihood of interaction. Here, edges were weighted by the inverse Euclidean distance between players u and v as follows:

(4) $$Weight\left(u,v\right)=\frac{1}{Distance\left(u,v\right)},$$

where u and v represent two players, and $Distance\left(u,v\right)$ is the Euclidean distance between their respective positions at a given second.

**Compute clustering coefficients from a graph**

The clustering coefficient $V_{i}^{\mathrm{clust},}$ for a node (player) i in a weighted graph is defined as follows:

(5) $$V_{i}^{\mathrm{clust}}=\frac{1}{k_{i}\left(k_{i}-1\right)}\;\sum_{j,k}\frac{w_{ij}+w_{ik}}{2}a_{jk},$$

where $k_{i}$ is the degree of node i, which represents the number of neighbors connected to node i, and $w_{ij}$ and $w_{ik}$ are the weights of the edges connecting node i to nodes j and k, respectively. The last term $a_{jk}$ is an indicator function, which is 1 if there is an edge between nodes j and k, and 0 otherwise.

The global clustering coefficient is then calculated by taking the average of individual clustering coefficients across all players within a team, as floolows:

(6) $$V^{\mathrm{clust}}=\frac{1}{N}\sum_{i=1}^{N}V_{i}^{\mathrm{clust}}$$

where N is the total number of players in the team.

Using the velocity- and distance-based edge calculations described above, we can derive two macroscopic features, as follows: $V^{clust,vel}$, clustering coefficient based on velocity similarity, and $V^{clust,dist}$ clustering coefficient based on inverse distance. These metrics capture different aspects of team coordination and spatial organization. NetworkX library in Python [21] was used in the implementation of the analysis.

### 2.3. Causal Emergence and Possession Rates

To investigate how team-level emergent dynamics relates to team performance, we examined the relationship between causal emergence and possession rates. The possession rate, defined as the percentage of time a team maintains control of the ball, is a pivotal metric often considered a decisive factor in determining match outcomes. By focusing on the differences between home and away teams, this analysis aimed to uncover the extent to which emergent team dynamics contribute to maintaining control of the ball, a key aspect of successful gameplay.

Causal emergence values were derived using macroscopic features based on player tracking data, including relative distance ($V_{t}^{\mathrm{dist}}$), CoM ($V_{t}^{\mathrm{CoM}}$), the clustering coefficient of inverse distance ($V_{t}^{\mathrm{clust},\;\mathrm{dist}}$), and velocity similarity ($V_{t}^{\mathrm{clust},\;\mathrm{vel}}$). For each match, the difference in average causal emergence values between the home and away teams was computed separately for the first and second halves. Similarly, the differences in possession rates between home and away teams were obtained for the same periods.

We correlated these paired differences (home vs. away) to assess the relationship between causal emergence and possession rates. Pearson’s correlation coefficient (R) and the corresponding *p*-values were used to quantify the strength and statistical significance of these relationships, providing insights into how emergent team dynamics is related to ball control.

### 2.4. Causal Emergence Before Shooting

To analyze causal emergence before shooting events, a sliding window approach was employed to segment and compare the emergence values over time. This method aimed to capture the dynamic coordination patterns leading up to shooting events.

For each shooting event, a 60-s interval of emergence data preceding the event was extracted. The window size of calculating each causal emergence was set to 60 s to ensure sufficient temporal resolution for identifying emergent patterns, while the step size was defined as 1 s to provide granular temporal alignment. The shooting event served as the temporal anchor (time-lock), allowing consistent alignment of emergence values across all analyzed segments. Specifically, the 60-s interval began exactly 60 s before the recorded frame of the shooting event and ended at the frame immediately preceding the shot.

To establish a baseline for comparison, random 60-s intervals were selected from the same half of the game where the shooting event occurred. These segments were chosen randomly within the start and end frames of each half. The baseline segments were generated using the same sliding window parameters (60-s window size and 1-s step size) to maintain consistency in data structure and temporal granularity.

Although the defending teams occasionally regained possession during the analyzed windows, the labels of the shooting teams were maintained for clarity and consistency. This approach allowed this study to focus on the emergent behaviors of the attacking team, regardless of temporary disruptions in ball possession rate.

For each match, emergence values were extracted from preprocessed positional data and aligned with shooting events. The shooting frames were identified using event data, which recorded match-specific actions, including passes, fouls, and shots. Frames corresponding to the start of the first half, the start of the second half, and their respective end frames were used to segment the emergence data. For each shooting event, the corresponding team and half were identified to ensure correct alignment between emergence values and event data.

The emergence values from shooting intervals and their respective baseline segments were aggregated for subsequent statistical analysis. This comparison provided insights into whether patterns of causal emergence differed significantly between shooting scenarios and randomly selected segments, revealing critical features of team coordination and dynamics leading to shots.

## 3. Results

### 3.1. Match-Level Analysis: Degree of Causal Emergence Correlates with Possession Rate

To investigate how the degree of causal emergence relates to team performance, we conducted a match-level analysis examining the relationship between causal emergence and possession rates. Possession rate, defined as the percentage of time a team controls the ball, is a pivotal metric often regarded as a decisive factor in determining match outcomes [22]. This analysis aimed to uncover the extent to which emergent team dynamics, as quantified by causal emergence, contribute to a team’s ability to maintain ball possession, a critical component of effective gameplay and overall success.

In this analysis, our objective was to understand how collective behaviors emerge in football teams by examining causal emergence in relation to the players’ positions and velocities. To represent team coherence at a higher level, we first used relative distance and CoM as macroscopic features, capturing the collective movement of players on the field. Previous studies have shown that CoM is an effective indicator of team coordination and collective behavior, especially in scoring zones where spatial organization and coordinated actions are crucial for success [23].

We examined the relationship between causal emergence and ball possession rate across the first and second halves of multiple matches. Specifically, causal emergence was calculated using the following macroscopic features: relative distance ($V_{t}^{\mathrm{dist}}$), CoM ($V_{t}^{\mathrm{CoM}}$), inverse distance ($V_{t}^{\mathrm{clust},\;\mathrm{dist}}$), and velocity ($V_{t}^{\mathrm{clust},\;\mathrm{vel}}$).

To account for the variability between the two halves of each match, we computed the differences in average causal emergence values and possession rates between the home and away teams for each half. This approach ensures a standardized comparison by mitigating the effects of game-specific conditions and strategic variations across halves.

**Figure 2:**
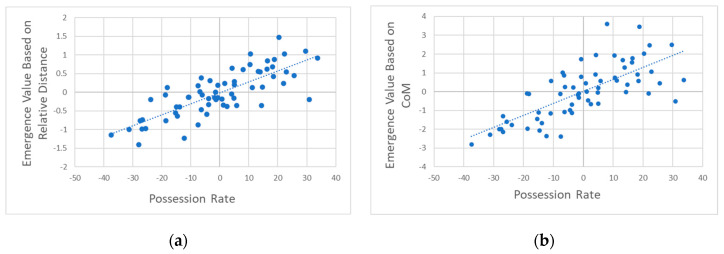
Correlation of differences in causal emergence based on aggregate positioning metrics and ball possession rates. (**a**) Causal emergence based on relative distance exhibits a high correlation with ball possession rate, with $R=0.79\;\left(p<0.001\right)$. (**b**) Causal emergence based on CoM shows a similar strong correlation with possession rate at $R=0.74\;\left(p<0.001\right)$.

In Figure 2, the results demonstrate a strong relationship between ball possession rate and causal emergence metrics derived from aggregate positioning metrics. Teams with higher causal emergence values indicative of greater synergistic team dynamics tend to achieve higher ball possession rates. This finding highlights that spatial structure, as captured by macroscopic features, plays a crucial role in facilitating ball retention. The high correlation $\left(R=0.79\right)$ observed with relative distance ($V_{t}^{\mathrm{dist}}$) as macroscopic features suggest that emergent team-level dynamics, as captured by relative positioning, play a critical role in maintaining possession. Teams in possession of the ball likely exhibit higher synergy, as their spatial arrangements reflect coordinated offensive structures that promote ball retention and passing options.

Causal emergence based on CoM ($V_{t}^{\mathrm{CoM}}$) also exhibits a strong correlation with the ball possession rate $\left(R=0.74\right)$, indicating that when a team possesses the ball, their macroscopic configuration reflects a higher level of coordinated interaction, or synergy, compared to the opposing team. This emergent synergy likely arises as the possessing team dynamically adjusts its structure to create passing lanes, maintain spacing, and responds to the opposing team’s defensive movements.

**Figure 3:**
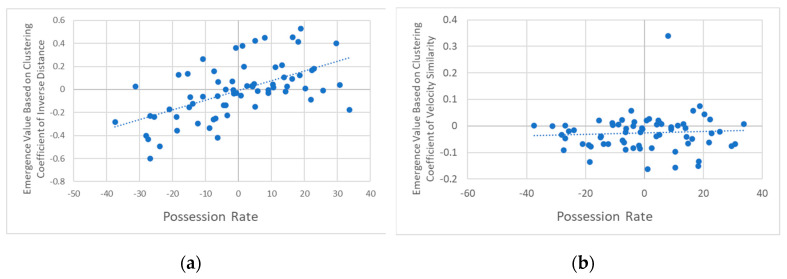
Correlation of differences in causal emergence based on network interaction metrics with ball possession rates. (**a**) The correlation between causal emergence based on the clustering coefficient of inverse distance and possession rate yielded a moderate correlation of $R=0.58\;\left(p<0.01\right)$. (**b**) The correlation between causal emergence based on clustering coefficient of velocity similarity and possession rate showed a near-zero correlation, with $R=0.06\;\left(p=0.71\right)$.

In Figure 3, causal emergence derived from the clustering coefficient of inverse distance ($V_{t}^{\mathrm{clust},\;\mathrm{dist}}$) demonstrated a moderate positive correlation with possession rate differences $\left(R=0.58\right)$. This result shows that higher $V_{t}^{\mathrm{clust},\;\mathrm{dist}}$, which reflects stronger local spatial interconnectedness within the team’s positional network, is associated with higher possession rates. This finding suggests that spatial arrangement plays a critical role in sustaining ball control.

In contrast, causal emergence derived from the clustering coefficient of velocity similarity ($V_{t}^{\mathrm{clust},\;\mathrm{vel}}$) showed no meaningful correlation with the ball possession rate $\left(R=0.06,\;p=0.71\;\right)$. This lack of significant correlation suggests that player velocity alignment alone is not strongly associated with the ball possession rate. While velocity synchronization might reflect localized tactical coordination, it does not appear to directly contribute to the team-level emergent dynamics required for sustained possession.

The analysis reveals that causal emergence, as quantified through various macroscopic features, especially from spatial organization, correlates strongly with possession rate, underscoring the critical role of emergent team dynamics in effective ball control. However, it also suggests that velocity alignment alone is insufficient to capture the emergent team dynamics necessary for maintaining ball control.

In the next section, we will focus on the critical event of shooting, examining the detailed changes in causal emergence leading up to pivotal moments.

### 3.2. Causal Emergence over Players’ Positions

In this analysis, we compared two macroscopic features based on player tracking data, including relative distance ($V_{t}^{\mathrm{dist}}$), and CoM ($V_{t}^{\mathrm{CoM}}$). For the relative distance calculation, we defined each player’s distance from the center of the field as the microscopic feature. The macroscopic feature was represented by the average distance of all players’ positions to the center of the field. This approach captures the spatial coherence of players relative to a fixed reference point (i.e., the center of the field), simplifying the representation of team dynamics.

**Figure 4:**
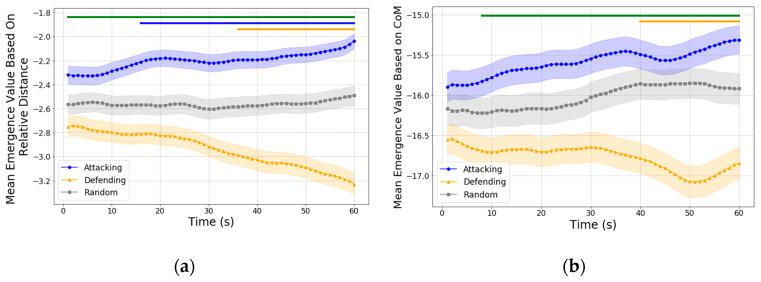
Causal emergence with (**a**) relative distance and (**b**) CoM as macro features within the 60 s leading up to a shot. Each data point represents the causal emergence calculated using a 60-s time interval ending at the point of measurement. Shaded regions indicate the standard error of the mean (SEM) for each data point across all events. The blue lines represent values for the team that took the shot (attacking team), while the orange lines represent values for the opposing team (defending team). The gray lines depict the average causal emergence values during randomly selected 60-s intervals, matched in number to the actual shooting events. This provides a baseline comparison between dynamics before shooting events and neutral or random phases of play. Colored ribbons on top of the graph indicate statistically significant differences between groups, with Bonferroni correction for multiple comparisons. The green ribbon denotes significant differences between the attacking team (blue line) and defending team (orange line). The blue ribbon indicates significant differences between the attacking team (blue line) and random intervals (gray line). The orange ribbon marks significant differences between the defending team (orange line) and random intervals (gray line).

Our analysis focused on evaluating causal emergence values before shooting, using relative distance and CoM as the macroscopic features. As shown in Figure 4, for both (a) relative distance and (b) CoM analyses, the attacking team exhibits an increasing trend in causal emergence values as the shot approaches, while the defending team shows a decreasing trend.

To quantitatively assess trends in causal emergence, we conducted linear regression analyses for each condition across both relative distance and CoM representations. For the relative distance analysis, for the attacking team, the regression analysis yielded a significant positive slope, $\beta =0.0035,\;R^{2}=0.822,\;p<0.001$, indicating a strong increase in causal emergence values over time. In contrast, the defending team exhibited a significant negative slope, $\beta =-0.0080,\;R^{2}=0.967,\;p<0.001$, indicating a significant decrease in causal emergence values. Random intervals serve as a baseline, showing flatter trends with smaller slope values than attacking and defending. For the random attacking intervals, the slope was $\beta =0.0004,\;R^{2}=0.037,\;p=0.139$, which does not display strong directional trends. This baseline comparison underscores that the increasing and decreasing trends in causal emergence are specific to shooting phases rather than non-shooting phases.

The increasing causal emergence in the attacking team suggests coordinated and synergistic movement patterns likely driven by structured offensive strategies as they progress toward a shot. Conversely, the defending team appears to react to the attackers, resulting in an increase in redundancy in the seconds leading up to the shot. This difference in causal emergence trends illustrates how team coordination evolves distinctly in offensive versus defensive contexts.

Similarly, for the CoM analysis (Figure 4b), the attacking team exhibited a significant positive slope, $\beta =0.0084,\;R^{2}=0.883,\;p<0.001$, indicating a strong upward trend in causal emergence values. The defending team showed a significant negative slope, $\beta =-0.0071,\;R^{2}=0.707,\;p<0.001$, reflecting a downward trend. However, unlike the relative distance, the random intervals in the CoM analysis also displayed slope values. For the random attacking intervals, the slope was $\beta =0.0076,\;R^{2}=0.848,\;p<0.001$. Although these intervals showed significant trends, they were not as pronounced as those observed during shooting phases.

These findings suggest that, while baseline spatial coordination is present in both teams, the emergence dynamics during shooting intervals are distinct, with the attacking team demonstrating the strongest positive trend in causal emergence and the defending team showing a clear negative trend.

Both the relative distance and CoM analyses reveal consistent trends, with increasing causal emergence in the attacking team and decreasing values in the defending team. This consistency across CoM representations confirms that CoM is an effective macroscopic feature for analyzing emergent behavior in team dynamics. The observed trends likely reflect coordinated offensive movements that rely on coherence actions to create scoring opportunities, while the defending team’s responses may result in a breakdown of coherence.

### 3.3. Clustering Coefficients as an Emergent Feature

#### 3.3.1. Causal Emergence of Clustering Coefficients Based on Velocity Similarity

We used clustering coefficients based on velocity similarity to analyze local coordination among players, focusing on the similarity in their movement velocities and directions. By examining velocity similarity, we aimed to understand how closely players align their movement patterns, which is essential for maintaining coordinated formations and responding to tactical demands. Connections between players were weighted based on velocity and directional similarity, providing a measure of how synchronized the players are in their movement as they approach key moments like a shot.

**Figure 5:**
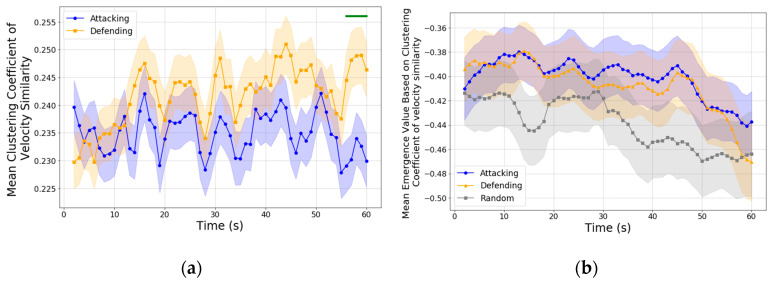
(**a**) Clustering coefficient of velocity similarity for the attacking team (blue) and defending team (orange) over the 60 s leading up to a shot. Shaded regions represent the standard error of the mean (SEM). While the defending team generally exhibits higher clustering coefficients, statistically significant differences were only observed in the final seconds before the shot, indicated by the green ribbon on top (α=0.05). (**b**) Causal emergence values based on clustering coefficients of velocity similarity over the same interval, capturing the emergent properties of velocity coherence.

In Figure 5, the clustering coefficients based on velocity similarity provide insights into the local coherence of player movements. Higher values indicate closer alignment in velocity and direction among neighboring players, often associated with a cohesive structure.

In terms of causal emergence, this analysis focuses on whether the dynamics of the clustering coefficient itself can be explained by microscopic features of velocity and direction, or if the clustering coefficient exhibits emergent properties that provide additional predictive power beyond the microscale. Both the attacking and defending teams exhibited a downward trend in causal emergence values over time. Specifically, the attacking team (blue) exhibited a mild downward trend with a slope of $\beta =-0.0024\;\left(R^{2}=0.091,\;p=0.019\right)$, and the defending team (orange) showed a similar downward trend with a slope of $\beta =-0.0028\;\left(R^{2}=0.109,\;p=0.010\right)$. Random intervals also displayed a downward trend, with slope of $\beta =-0.0010\;\left(R^{2}=0.742,\;p<0.001\right)$.

This decline in causal emergence values suggests that, as the shot approaches, the dynamics of the clustering coefficient based on velocity similarity become increasingly predictable by the microscopic features (velocity and direction). In other words, local coherence in velocity aligns more closely with individual-level movement patterns, indicating a shift from synergistic, emergent contributions toward more constrained and predictable movement patterns. This trend likely reflects the tactical adjustments made by both teams, as follows: the attacking team seeks to maximize forward momentum, while the defending team adopts structured formations to counteract offensive advances. As a result, the clustering coefficient’s predictive power becomes more tied to individual movement, limiting its emergent properties.

Interestingly, this trend in causal emergence based on velocity similarity contrasts with findings from the analysis of positional coherence, where causal emergence values for the attacking team increased as they approached the shot. This difference suggests that positional adjustments, compared to velocity alignment, may offer greater potential for emergent team-wide dynamics.

Unlike velocity similarity, which primarily supports immediate and localized coordination, positional coherence facilitates long-term strategies and anticipates complex interactions across the team. Thus, positional coherence can be considered better in terms of its ability to capture emergent behaviors that are not directly reducible to microscopic features. These emergent patterns likely play a critical role in creating scoring opportunities by leveraging the collective spatial dynamics of the team, a feature that is less pronounced in velocity-based analyses.

#### 3.3.2. Causal Emergence of Clustering Coefficients Based on Inverse Distance

To understand how spatial coordination contributes to team behavior during critical moments, we used player positions as the microscopic features and calculated clustering coefficients based on the inverse of the distances between players. This approach highlights how close positioning among players contributes to collective team dynamics. Previous studies have shown that, in team sports, spatial arrangement and compactness play a crucial role in enabling cohesive defensive or offensive structures, which directly impact emergent collective behaviors during crucial phases of play. By using the inverse distances among players, we can assess how tightly aligned or dispersed each team is, providing insights into the coordination level within the team.

**Figure 6:**
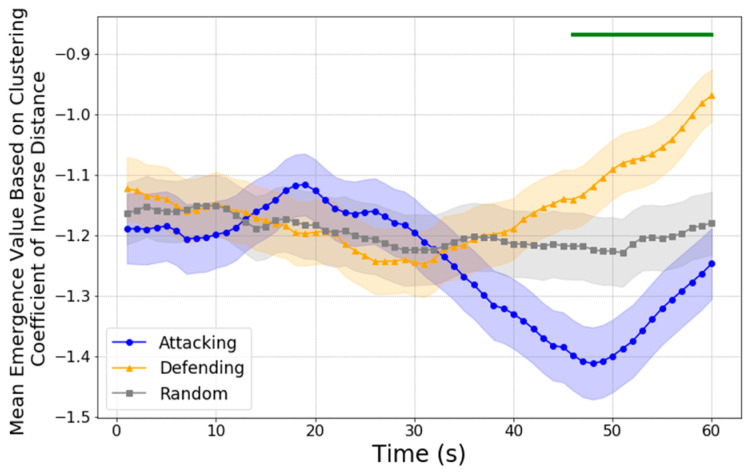
Causal emergence values with clustering coefficients based on inverse distances within the 60 s leading up to a shot. Shaded regions represent the standard error of the mean (SEM). Statistical significance is indicated by the green ribbon on top, which marks significant differences between the attacking team (blue line) and defending team (orange line) from the 45-s mark onward (Bonferroni corrected). Linear regression analysis for the last 30 s of the defending team suggests a consistent upward trend in causal emergence. For the attacking team, analysis over the last 10 s shows a steep increase in causal emergence just before the shot.

As shown in Figure 6, the causal emergence values for both teams exhibit discernible patterns over the 60 s before a shot. In the first 30 s, neither team’s causal emergence deviates significantly from the values observed in randomly selected intervals, suggesting that their positioning dynamics do not substantially enhance overall emergent behavior during this initial period.

After the 30-s mark, the defending team begins to show a marked increase in causal emergence, statistically exceeding that of the attacking team from approximately the 40-s mark onward (indicated by the green ribbon on top). The upward trend in the defending team’s causal emergence, as confirmed by the linear regression results with slope $\beta =0.0089,\left(R^{2}=0.976,\;p<0.001\right)$, indicates that their positioning dynamics become increasingly predictive of team-level coherence. This increase likely reflects their effort to maintain a compact defensive structure as they adapt their positioning to counteract the attacking team’s movements. The higher causal emergence values suggest that the collective positioning of defending players becomes more tightly aligned, creating an emergent pattern that enhances their ability to block potential shooting opportunities and maintain spatial control.

For the attacking team, a noticeable increase in causal emergence only appears within the last 10 s before the shot, as indicated by the steep slope from the linear regression where slope $\beta =0.0158,\left(R^{2}=0.998,\;p<0.001\right)$. This dynamic follows a U-shaped trend, where causal emergence values initially decrease during the earlier phase of the 60-s window before rising sharply in the final moments. This U-shaped pattern reflects a shift in the attacking team’s spatial dynamics, as follows: the initial dip may indicate a period of spatial adjustment as players transition from broader formations into more focused attacking configurations. The late surge in causal emergence suggests that players achieve a level of spatial coherence characterized by strategic positioning that maximizes scoring opportunities.

In summary, the causal emergence values based on inverse distances reveal that the defending team enhances their spatial coherence earlier, responding proactively to the attacking threat, while the attacking team’s emergent behavior becomes more pronounced only in the critical seconds before a shot. This pattern underscores the tactical adjustments made by each team, with the defending team maintaining compact formations to limit scoring opportunities and the attacking team intensifying their positional coherence to maximize goal-scoring potential in the final moments.

## 4. Discussion

### 4.1. Discussion About the Current Result

This study applies the concept of causal emergence to the analysis of football match dynamics, providing a novel framework to understand how team-level behaviors emerge from individual player interactions. To examine different aspects of team collective behavior, we derive causal emergence using the following four different macroscopic features: relative distance, CoM, clustering coefficient of inverse distance, and velocity similarity.

When relative distance ($V_{t}^{\mathrm{dist}}$) and center of mass ($V_{t}^{\mathrm{CoM}}$) were used as macroscopic features, both showed strong positive correlations between casual emergence and ball possession rate. The findings imply that teams with higher causal emergence, which manifested through well-structured relative positions and collective movements, are better equipped to sustain possession. This observation could result from higher player cohesion than opposite team, or adaptive in-game decision making. In the context of shooting dynamics, causal emergence derived from $V_{t}^{\mathrm{dist}}$ and $V_{t}^{\mathrm{CoM}}$ exhibited similar trends, as follows: for attacking teams, causal emergence increased significantly as they approached a shot, reflecting improved coordination and spatial organization during offensive phases. Conversely, for defending teams, a decline in causal emergence was observed for both features, indicating an increase in redundancy at the micro level. This suggests that defending teams rely more on synchronized and predictable actions among players to maintain stability in defensive formations. These results highlight the distinct dynamics between offensive and defensive phases, demonstrating how macro-level spatial coherence relates to team strategies and possession outcomes.

When clustering coefficients derived from inverse distance ($V^{clust,dist}$) were used as the macroscopic feature, a moderate positive correlation between casual emergence and ball possession rate was observed. This suggests that localized spatial formations contribute to higher-level emergent dynamics that are critical for sustaining possession than that of the opposite team. This reflects the ability of teams to integrate local proximities into cohesive, system-wide behaviors that enhance ball control and adaptability during gameplay. In the context of shooting dynamics, causal emergence for the defending team showed a steady increase after the 30-s mark. For the attacking team, a sharp rise in causal emergence occurred in the final 10 s before the shot, contrasting with the relatively stable values observed earlier.

For clustering coefficients derived from velocity similarity ($V^{clust,vel}$), no significant correlation with ball possession rate was found. Additionally, before shooting events, velocity-based clustering coefficients showed minimal variation. These findings suggest that player velocity alignment, while reflecting localized synchronization during specific tactical moments (e.g., attacking from own half or counterattacks), does not strongly contribute to emergent, system-level dynamics critical for sustained possession or defensive coordination in pivotal moments. This lack of correlation implies that velocity similarity alone may capture short-term, transient tactical behaviors rather than long-term or stable emergent patterns integral to overall team strategy. In the context of possession, spatial organization (as reflected in relative distances, CoM, or clustering based on inverse distance) may play a greater role in team coherence and emergent dynamics than simple velocity alignment.

Taken together, these findings reveal distinct patterns of causal emergence across different macroscopic features, highlighting spatial coherence as a key driver of emergent team dynamics during possession and shooting phases, while underscoring the limitations of velocity-based metrics in capturing the complexity of team strategies. More broadly, it demonstrates the value of causal emergence as a framework for analyzing complex, real-world systems where interdependent components interact to produce collective behaviors. This research thus establishes a foundation for using causal emergence as a new metric in sports analytics, offering coaches and analysts an innovative perspective on team coordination and game strategies.

### 4.2. Link to Other Literature

From the perspective of theoretical frameworks of emergence, this study highlights the dynamic interplay between synergy and redundancy in shaping team-level behaviors. Previous works from Hoel et al. [13] and Rosas et al. [15] have formally shown that macro-level properties often provide additional predictive power that cannot be fully explained by micro-level components alone. However, these studies primarily emphasized synergistic phenomena, focusing on how macro-level coordination emerges from micro-level interactions. Our analysis considers the dynamics between redundancy and synergy, a factor critical in real-world environments like football games. In the context of football, redundancy is inherently present, as players must maintain a degree of synchronization to form cohesive attacking or defensive structures. Synergy could emerge when individual player contributions combine in non-linear ways to enhance overall team effectiveness. Our findings suggest that the interplay between these two forces dynamically shifts, with attacking teams demonstrating higher causal emergence metrics before shooting events, reflecting a transition from redundancy-dominated formations to synergy-driven coordination.

By integrating these theoretical principles into the analysis of team sports, our study provides a practical application of causal emergence. It demonstrates how macro-level coordination, quantified through causal emergence metrics, contributes to an understanding of coordinated human behaviors in high-pressure, competitive environments.

Our findings offer a perspective that complements previous research emphasizing the role of team dynamics in sports science. Duarte et al. highlighted that irregularities in individual player synchronization can contribute to stabilizing team-level coordination by allowing localized variability to support collective behavior [1]. This concept aligns with our observation of increasing causal emergence derived from relative distance and the center of mass (CoM) for attacking teams before shooting. Our results suggest that these emergent properties may reflect how localized spatial adjustments among players enhance macro-level coordination, thereby facilitating effective offensive actions.

Another possible interpretation of the observed differences in causal emergence based on aggregate positioning metrics between attacking and defending teams is the distinction of exploratory and exploitation [24]. We could consider attacking as a process of exploration, where players adjust their positioning and passing to identify potential weaknesses of the opponents to attack. On the other hand, defensive strategies, in contrast, often exhibit a more exploitative structure, as teams rely on pre-established defensive formations and strategies.

For the low correlation of possession rate and causal emergence based on clustering coefficients, one possible way of considering it is the constraints of human coordination capacity. Studies suggest that human groups typically coordinate most effectively within subgroups of approximately five to six individuals, beyond which coordination becomes increasingly difficult without a hierarchical structure or simplified decision-making processes [25,26,27]. In the context of football, this limitation may explain why localized coordination measured by clustering coefficients at the whole team level does not strongly correspond to possession rate. To better capture the role of hierarchical and subgroup-based coordination, future studies could consider partitioning players into functional subgroups based on their roles (i.e., defenders, midfielders, and forwards) and then calculating causal emergence within these units.

One behavioral interpretation of why the field center plays a crucial role in the causal emergence of team coordination in our analysis is that it acts as a naturally emergent spatial structure that shapes collective movement. This concept aligns with stigmergy, where individuals coordinate indirectly by interacting with modifications in their environment rather than through direct communication [28]. In football, the field center provides a shared spatial reference that influences team formations and movement patterns. Players dynamically adjust their positioning in response to their teammates, opponents, and their relation to the field center, creating coordinated movement patterns. This self-organized process enables teams to maintain coherence without constant direct communication, emphasizing the significance of macro-level spatial features in team dynamics.

From a team behavior perspective, negative values of causal emergence $\left(\Psi <0\right)$ may offer additional insights into the learning and adaptation processes underlying team coordination. Specifically, negative Ψ values indicate that individual-level information redundancy outweighs macro-level predictive power. The behavioral interpretation of this phenomenon remains open. One possible explanation for this is that these negative values reflect the players’ learning and adaptation, leading to localized synchronization rather than emergent team-wide coordination. This aligns with research on team learning and coordination, which suggests that high-performing teams develop shared cognitive models through repeated interaction rather than relying solely on individual skill diversity [29,30]. Future research could further investigate the relationship between information redundancy, synchronization, and adaptive team behavior.

Recent studies have further explored the application of collective behavior metrics in team sports. For example, a collective movement analysis demonstrated how coordination tactics among football players emerge through spatiotemporal dynamics during gameplay [31]. Our findings align with this perspective by showing how spatial metrics like relative distance and CoM reveal emergent team behaviors that are critical for sustaining possession and enhancing offensive coordination. Additionally, a systematic review of collective tactical behaviors in football using positional data highlighted the importance of spatial metrics, such as player distance and movement patterns in understanding team performance [32]. Our work builds on this foundation by explicitly linking these spatial metrics to emergent team dynamics, demonstrating their relevance not only for performance evaluation, but also for understanding distinct offensive and defensive strategies.

Research in network science applied to football and water polo has demonstrated that passing interactions among players play a crucial role in performance outcomes [33,34]. While these studies focus on static passing-based edges, our approach utilizes a moving window of seconds to calculate clustering coefficients, forming dynamic graphs that evolve continuously throughout gameplay. This method captures continuous spatiotemporal interactions among players, providing a more detailed view of how emergent team dynamics change during match events. Additionally, multilevel hypernetworks have been employed to analyze team synergies, offering a comprehensive framework to quantify interactions across different levels of complexity. This approach highlights how tactical configurations, local interactions (e.g., two vs. one scenarios), and key player contributions collectively shape team dynamics, providing valuable insights into the emergent properties of competitive team performance [35]. Studies in rugby have also investigated the emergence of collective states, demonstrating how defensive formations exhibit higher order and compactness compared to the more dispersed and dynamic structures during attacks, thereby highlighting the universality of collective behavior metrics across different team-based activities [36]. Our result aligns with this perspective, as we also observed that clustering coefficients derived from velocity similarity were higher during defensive phases than offensive phases. This suggests that defensive strategies may prioritize synchronized and compact formations, while offensive behaviors rely more on dynamic spatial organization. These parallels highlight the relevance of collective behavior metrics across sports, offering a shared framework to explore emergent team dynamics.

Our findings also align with the broader perspectives on team synergies and collective behavior in sports science. Araújo and Davids emphasized the theoretical framework of team synergies, describing them as emergent properties arising from interactions within the team [37]. Similarly, the concept of sports teams as superorganisms emphasizes how individual contributions and interactions within a team operate similarly to biological systems, such as ant colonies or bee hives, where collective behaviors arise from local interactions [38].

By integrating these perspectives, our study builds on existing frameworks while providing a novel approach to analyzing macro-level team strategies through causal emergence metrics. This research underscores the potential of these metrics to provide deeper insights into team coordination, possession strategies, and broader collective dynamics in football.

### 4.3. Limitations

This study primarily focused on intra-team interactions, examining how individual players’ behaviors contribute to macro-level team dynamics. However, in competitive environments, emergent behaviors within one team are heavily influenced by inter-team interactions. The opposing teams’ strategies and spatial arrangements can significantly shape the dynamics of play, particularly during critical phases such as transitions or set plays. Future research should integrate inter-team dynamics to better capture the mutual influences and adaptive strategies between competing teams.

Our analysis considers individual players as the first-order micro-level components and examines how their interactions contribute to macro-level team behaviors. However, we do not explicitly account for higher-order dynamics like the combined influence of multiple players or interactions within subgroups. Rosas et al.’s framework for causal emergence includes higher-order interactions, such as pairwise or subgroup-level combinations, as micro-level contributors [15]. By focusing solely on individual players versus the whole team, our approach may overlook the interdependencies that arise from these higher-order structures, potentially underestimating the complexity of team coordination. Extending causal emergence metrics to include these higher-order interactions would provide a more comprehensive understanding of team dynamics.

Another limitation is that the causal emergence metrics employed in this study provide relative comparisons between synergy and redundancy, but they do not yield absolute measures of these quantities. This limitation restricts the application of such metrics for cross-contextual comparisons, such as benchmarking team performance across different contexts, matches, or sports. Developing absolute measures of synergy and redundancy is an active area of research, particularly within the framework of Partial Information Decomposition (PID) [16]. By adopting or extending PID methodologies, future research could establish absolute metrics for causal emergence, enabling robust cross-contextual analyses and advancing the generalizability of causal emergence as an analytical framework for team dynamics.

### 4.4. Future Directions

The study of causal emergent team dynamics in sports presents several directions for future research. One important direction involves the integration of machine learning techniques to model and predict emergent behaviors based on spatiotemporal player data. Advanced algorithms, such as neural networks and graph-based models, could uncover hidden patterns in player interactions, providing deeper insights into how micro-level behaviors scale into team-level dynamics. These methods would enable the development of predictive models that enhance the understanding of emergent behaviors under various tactical and environmental conditions.

Another critical area for exploration is the application of causal emergence metrics across different sports contexts. Sports such as rugby, basketball, and water polo exhibit distinct spatiotemporal and interaction dynamics compared to football. Comparative analyses could validate and refine causal emergence methodologies, identifying universal principles of collective coordination and adaptation. These studies would also highlight context-specific variations, offering a broader perspective on the role of emergent dynamics in diverse team-based activities.

Temporal analysis of emergent dynamics is another valuable direction. Understanding how causal emergence evolves throughout matches, particularly during critical phases such as transitions between offense and defense, high-pressure situations, or set plays could provide a richer understanding of team adaptability. Longitudinal studies that track these dynamics over entire games or seasons would elucidate how emergent properties evolve and stabilize under changing contexts, contributing to the development of time-sensitive models of team performance.

## 5. Conclusions

This study employs causal emergence to investigate collective team dynamics in football, analyzing macroscopic features derived from player tracking data to quantify how collective behaviors emerge from individual interactions. The findings demonstrate that causal emergence successfully captures team coordination, exhibiting a strong correlation with ball possession rates and increased emergence during attacking phases. These results suggest that collective spatial organization and coherence are required for successful offensive actions.

The results identify key distinctions in the mechanisms driving offensive and defensive team behaviors. Offensive strategies leverage synergy to achieve coordinated movements and exploit spatial opportunities, while defensive strategies rely more on redundancy to maintain structural stability. The use of causal emergence as a quantitative framework provides a detailed method for linking micro-level interactions with macro-level team dynamics

These findings advance our understanding of the role of emergent dynamics in shaping team performance and strategies.

## Data Availability

The dataset analyzed in this study was provided by DataStadium Inc. under a licensing agreement and is not publicly accessible. Any requests for data access are subject to the terms and conditions set by DataStadium Inc.
